# Improved outcomes with leadless vs. single-chamber transvenous pacemaker in haemodialysis patients

**DOI:** 10.1093/europace/euae257

**Published:** 2024-10-01

**Authors:** Alexandre Panico, Adrien Flahault, Francis Guillemin, Emilie Varlet, Cécile Couchoud, Marc Bauwens, Eloi Marijon, Stéphane Roueff, Hélène Lazareth

**Affiliations:** Department of Nephrology, Centre Hospitalier Régional Universitaire de Nancy, Rue du Morvan, 54500 Vandoeuvre-Les-Nancy, France; Centre Hospitalier Régional Universitaire de Nancy, Inserm, Université de Lorraine, Clinical Investigation Centre—Clinical Epidemiology, Nancy, France; Department of Nephrology, Centre Hospitalier Régional Universitaire de Nancy, Rue du Morvan, 54500 Vandoeuvre-Les-Nancy, France; Université de Lorraine, Inserm, UMR INSPIIRE, 9 avenue de la Forêt de Haye, 54500 Vandoeuvre-Les-Nancy, France; Centre Hospitalier Régional Universitaire de Nancy, Inserm, Université de Lorraine, Clinical Investigation Centre—Clinical Epidemiology, Nancy, France; Université de Lorraine, Inserm, UMR INSPIIRE, 9 avenue de la Forêt de Haye, 54500 Vandoeuvre-Les-Nancy, France; Department of Cardiology, APHP, Hôpital Européen Georges Pompidou, Paris, France; REIN Registry, Agence de la biomédecine, Saint Denis La Plaine, France; REIN Registry, Agence de la biomédecine, Saint Denis La Plaine, France; Department of Nephrology, Centre Hospitalier Universitaire de Poitiers, Poitiers, France; Department of Cardiology, APHP, Hôpital Européen Georges Pompidou, Paris, France; Faculté de Médecine, Université Paris Cité, Paris, France; Paris Cardiovascular Research Centre, INSERM UMR-S 970, Paris, France; Department of Nephrology, APHP, Hôpital Européen Georges Pompidou, Paris, France; Faculté de Médecine, Université Paris Cité, Paris, France; Department of Nephrology, APHP, Hôpital Européen Georges Pompidou, Paris, France

**Keywords:** Leadless pacemakers, Single-chamber transvenous pacemakers, Haemodialysis, Survival, Complications, Vascular access for haemodialysis

## Abstract

**Aims:**

Cardiac conduction disorders are common in haemodialysis patients, with a relatively high rate of pacemaker implantations. Pacemaker-related complications, especially lead infections and central venous stenosis, pose significant challenges in this population. This study aims to compare single-chamber leadless pacemaker to single-chamber transvenous pacemakers in terms of survival and related complications in haemodialysis patients.

**Methods and results:**

This retrospective study included adult haemodialysis patients who received a first single-chamber transvenous or leadless pacemaker between January 2017 and December 2020. Data were obtained from the French national REIN registry matched to the national health databases (Système National des Données de Santé). Propensity score matching was used to balance baseline characteristics. Survival and complications were compared between groups by Cox regression and by competitive risk models, respectively. One hundred and seventy-eight patients were included after propensity score matching, with 89 patients in each group. The median follow-up time was 24 (range 7–37) months. Leadless pacemakers were associated with significantly lower all-cause mortality rates compared to transvenous pacemakers [hazard ratio (HR) = 0.68, 95% confidence interval (CI) (0.47–0.99)]. Device-related infections are significantly lower with leadless pacemakers throughout the follow-up period (HR 0.43, 95% CI 0.21–0.86). Leadless pacemaker recipients also required fewer vascular access interventions [odds ratio 0.53, 95% CI (0.33–0.68)] on arteriovenous fistula.

**Conclusion:**

With the limitations of its observational design, this study suggests that leadless pacemakers are associated with a lower rate of complications and better survival as compared with transvenous VVI pacemakers in haemodialysis patients, supporting to consider their preferential use in this population.

## Structured Graphical Abstract

**Structured Graphical Abstract:**
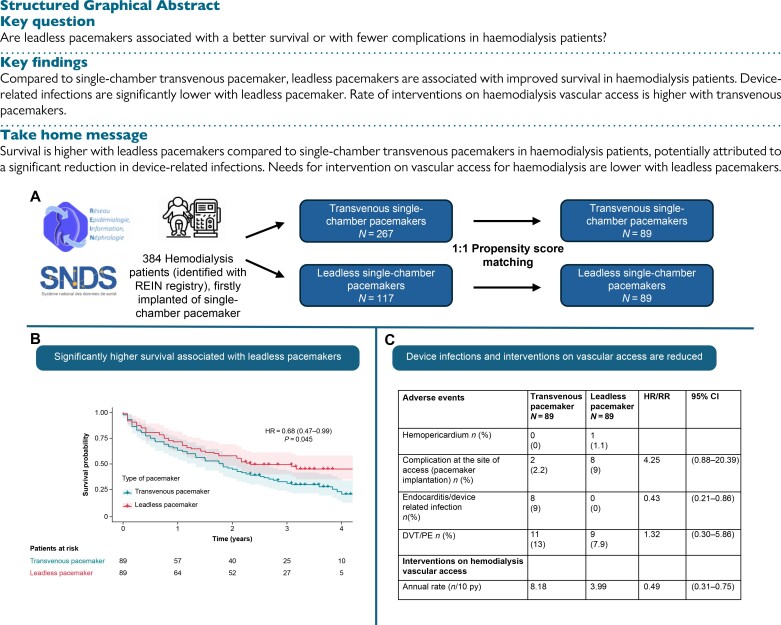
Outcomes of haemodialysis patients implanted with a leadless pacemaker compared to single-chamber transvenous pacemaker. (*A*) The patient enrolment process and the constitution of propensity score-matched sample. (*B*) A comparison of survival between leadless and transvenous pacemakers in haemodialysis patients. (*C*) A comparison of adverse events related to pacemaker implantation between groups. 95% CI, 95% confidence interval; HR, hazard ratio; RR, relative risk; DVT/PE, deep vein thrombosis/pulmonary embolism.

**What’s new?**

- No comparative analysis between leadless pacemakers and single-chamber transvenous pacemakers had previously been specifically conducted in haemodialysis patients.
- Compared to single-chamber transvenous pacemaker, leadless pacemakers are associated with improved survival in haemodialysis patients.
- Device-related infections are significantly lower with leadless pacemaker.
- Rate of interventions on haemodialysis vascular access is higher with transvenous pacemakers.

## Introduction

Cardiac rhythm and conduction disorders are common comorbidities in haemodialysis patients,^1,2^ which explains the high prevalence of pacemaker implantation in this population (∼5% of patients). The need for a pacemaker is up to five times higher in haemodialysis patients compared to patients without chronic kidney disease.^3^ The frequency of pacemaker-related complications is also increased in haemodialysis patients compared to patients without end-stage kidney disease (ESKD)^4^ and remains time dependent.^5^

Pacemaker lead infection represents a serious complication, with mortality rates reaching up to 75% among haemodialysis patients.^6,7^ The high incidence of episodes of bacteraemia could trigger the high frequency of lead infection.^8,9^

Central venous stenosis is another pacemaker-related complication, occurring due to the transvenous approach used for pacemaker implantation. Routine screenings detect such stenosis in up to one-quarter of patients with transvenous pacemakers.^8^ While central venous stenoses are often asymptomatic in non-ESKD patients, they represent a challenge in haemodialysis by impacting arteriovenous fistula (AVF) haemodynamics and thus the quality of dialysis sessions. These stenoses may require interventional management.^9^

Single-chamber leadless pacemakers have been recently developed^10^ and have been approved for commercial use in France in 2016. This device is the result of ongoing advancements in technology and growing expertise in the field of pacing.^11^ They benefited from preferential reimbursement by social security in haemodialysis patients, with expected moderate improvement in service (Class III according to the French National Authority for Health—HAS).^12^ While the safety and efficacy of this device have been demonstrated in clinical observational studies, only a few comparative analyses have been conducted against single-chamber transvenous pacemakers. In the general population, leadless pacemakers were associated with a lower complication rate during follow-up, especially no infection.^13–17^ One observational study conducted in the USA focused on leadless pacemaker implantation in haemodialysis patients, recording good safety and no device-related infections in this high-risk population.^18^ Another observational study compared outcomes on subgroups at high-risk, including haemodialysis patients, with a 2-year follow-up. Reduction of complications and reinterventions among patients implanted with a leadless pacemaker was demonstrated in most subgroups, but not in ESKD patients.^19^ Authors assumed that this lack of difference could be explained by the high number of ESKD patients who received a leadless pacemaker compared to those who received a transvenous pacemaker, resulting in difficulties in achieving balance between the groups even with the application of propensity score (PS) weighting. Furthermore, this study included haemodialysis patients and other ESKD patients such as CKD Stage 5, patients undergoing peritoneal dialysis, or transplant recipients. The diagnosis of CKD was made either using Medicare enrolment data or the ICD-10 classification and codes, which could result in misdiagnosis. To date, no other comparative analysis between leadless pacemakers and single-chamber transvenous pacemakers has been specifically conducted in haemodialysis patients.

The primary objective of our study was to compare the survival in haemodialysis patients who received single-chamber transvenous pacemaker or a leadless pacemaker implantation. Secondary objectives included a comparative analysis of acute (≤90 days) and long-term complication rates associated with single-chamber transvenous vs. leadless pacemakers.

## Methods

### Study design and population

**Figure 1:**
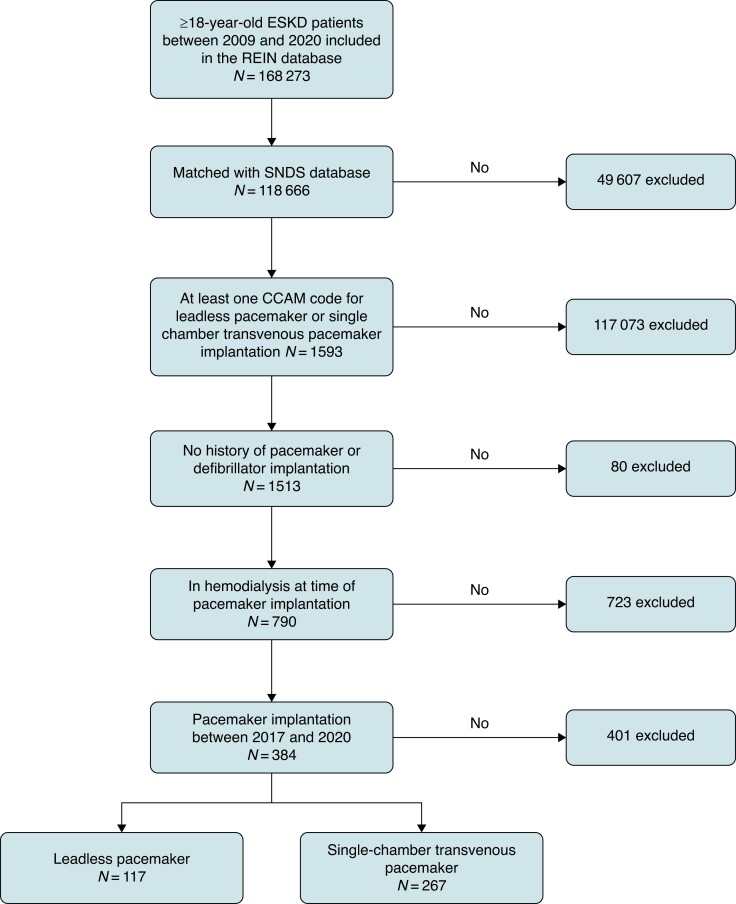
Flowchart illustrating the study protocol for patient enrolment. Out of 168 273 ESKD patients aged ≥18 years recorded in the REIN database between 2009 and 2020, 118 666 were successfully matched with the SNDS database. Subsequently, 1593 patients received at least one leadless or single-chamber transvenous pacemaker. Among them, 80 patients were excluded due to a previous history of cardiac electronic devices, 732 were not on dialysis at the time of pacemaker implantation, and 406 were excluded because the implantation occurred before the availability of leadless pacemakers (before 2017). This resulted in a final cohort of 384 patients. REIN, Réseau Epidémiologique et Information en Néphrologie; SNDS, Système National des Données de Santé; CCAM, Common Classification of Medical Acts.

All ≥18-year-old ESKD patients between 2009 and 2020 were identified in Réseau Epidemiologie et Information National (REIN) database, the French national registry that collects data regarding the socio-demographic characteristics, medical history, medical management, and events (i.e. transplantation and death) of all French ESKD patients.^20^ However, it does not contain specific information on pacemaker-related complications, which is available in the Système National des Données de Santé (SNDS) database, the French national health database that has collected data on the French population dating back to 2006. It collects data on hospital activity (i.e. inpatient and outpatient stay, diagnoses, procedures, and length of stay) for each patient hospitalization. Patients identified in REIN database were matched to the SNDS database using an iterative deterministic record linkage procedure.^21^ Unmatched patients were excluded from the analysis. After cross-referencing databases, we included in the analysis all haemodialysis patients who underwent the implantation of either a first single-chamber transvenous pacemaker or a leadless pacemaker between 1 January 2017 and 31 December 2020 (*Figure 1*). Exclusion criteria included a history of any other pacemaker or defibrillator implantation.

### Baseline comorbidities and encounter characteristics

Baseline patient characteristics were ascertained using the last available data in the REIN database. Baseline characteristics included age at pacemaker implantation, sex, body mass index (BMI), kidney disease responsible for ESKD, duration on dialysis, and vascular access for haemodialysis at the time of pacemaker implantation (i.e. AVF and catheter). Comorbidities were ascertained using the last available data in the REIN database coupled with the last available data in the SNDS database. Comorbidities included chronic heart failure, atrial fibrillation or flutter, complete atrioventricular block, other blocks, sinus dysfunction, coronary artery disease, ventricular or supraventricular arrhythmia, pulmonary hypertension, tricuspid valve disease, aortic aneurysm, peripheral arteritis, history of stroke, diabetes, and history of any type of cancer. ICD-10 codes used for identifying comorbidities are presented in Supplementary material (see Supplementary material online, *Table S1*).

### Characteristics of pacemaker implantation

The type of pacemaker and the implantation date were identified using the Common Classification of Medical Acts (CCAM) code available in the SNDS database (see Supplementary material online, *Table S1*). We also collected an identification code associated with the institution that performed the pacemaker implantation

### Outcomes

Our primary outcome was survival following pacemaker implantation throughout the study follow-up period. The censoring date was either the date of death, the date of kidney transplantation (which led to haemodialysis discontinuation), or 28 February 2023, which marks the last day of available data on patient survival. For secondary outcomes, we investigated acute complications within the first 90 days after implantation, including cardiac arrest, haemopericardium, pneumothorax or haemothorax, haemorrhage, vascular injury, deep vein thrombosis (DVT) or pulmonary embolism (PE), and device-related infection motivating or occurring during hospitalization. Additionally, we examined the length of hospital stay during the initial hospitalization when pacemaker implantation was performed. Infectious and thrombotic complications that occurred during the first 90 days were included in both acute and long-term complications. The censoring date for complications was defined as the earliest of the following: the date of death, the date of transplantation, or 31 December 2020, which marks the last day of available data from the SNDS database at the time of data extraction. Additionally, we examined interventions (endovascular procedures, surgeries) on dialysis access, the type of interventions performed, and the annual rate of interventions between both groups. The CCAM used for interventions and ICD-10 codes used for complications are available in Supplementary material online, *Appendix S1* (see Supplementary material online, *Table S1*).

### Statistical analysis

Baseline characteristics were compared with Wilcoxon’s tests for continuous variables and Fisher’s exact tests for categorical variables. The data set was complete, only for BMI that had a few missing values (*n* = 23, 5.9%), which were imputed using the median value. Balance was also assessed using standardized mean or proportion differences (SMD/SPD) where values exceeding 0.15 suggested imbalance between groups.^22^

To take into account differences in baseline characteristics, we used a PS matching to construct a matched sample where patients varied according to pacemaker type but were similar in terms of other confounders.^23^ The PS estimates the probability of being exposed to a leadless pacemaker based on baseline characteristics. It was estimated using a logistic regression model that included all socio-demographic characteristics, potential confounders, and all variables with a SMD/SPD >0.15 in absolute value.^24,25^ Each subject was assigned a probability to receive the leadless pacemaker. Subsequently, we conducted a 1:1 matching based on PS between the leadless pacemaker and single-chamber transvenous pacemaker groups using the nearest neighbour method with a calliper of 0.2 SD of the PS.^26^ Balance in baseline characteristics was assessed by performing a comparison of SMDs after matching (*Table 1*). Sensitivity analysis balancing covariates using an inverse probability of treatment weighting (IPTW) method was conducted.

**Table 1:** Baseline characteristics and comorbidities in overall sample and in paired sample after propensity score matching

	Overall sample				Paired sample			
Characteristics	Transvenous pacemaker, *n* = 267	Leadless pacemaker, *n* = 117	SMD/SPD	*P*-value	Transvenous pacemaker, *n* = 89	Leadless pacemaker, *n* = 89	SMD/SPD	*P*-value
Age in years, mean (SD)	80.2 (8.4)	75.5 (10.8)	−0.44	<0.001	77.9 (8.6)	77.3 (9.8)	−0.06	0.83
Age in years, median (IQR)	82 (75–86)	78 (71–83)			80 (74–84)	79 (73–84)		0.63
Female sex, *n* (%)	77 (29)	41 (35)	0.13	0.23	27 (30)	31 (35)	0.09	
BMI (kg/m^2^) mean (SD)	27.23 (5.83)	28.45 (5.42)	0.24	0.026	27.63 (5.93)	27.97 (4.88)	0.07	0.37
BMI (kg/m^2^) median (IQR)	26.49 (23.01–31.02)	28.21 (25.25–31.48)			26.54 (24.09–31.44)	28.03 (25.19–31.25)		
Missing	16	7			0	0		
Kidney disease, *n* (%)				0.038				0.41
Vascular nephropathy	88 (33)	25 (21)	−0.28		23 (26)	19 (21)	−0.11	
Diabetes	72 (27)	40 (34)	0.15		30 (34)	32 (36)	0.05	
Unknown	59 (22)	18 (15)	−0.19		18 (20)	11 (12)	−0.24	
Other	27 (10)	21 (18)	0.20		12 (13)	16 (18)	−0.24	
Glomerulonephritis	14 (5.2)	8 (6.8)	0.06		5 (5.6)	6 (6.7)	−0.04	
Polycystic kidney disease	7 (2.6)	5 (4.3)	0.08		1 (1.1)	5 (5.6)	0.20	
Vascular access for dialysis, *n* (%)				0.34				0.22
Arteriovenous fistula	180 (67)	81 (69)	0.04		67 (75)	59 (66)	−0.19	
Catheter	77 (29)	35 (30)	0.02		19 (21)	29 (33)	0.23	
Other	10 (3.7)	1 (0.9)	−0.31		3 (3.4)	1 (1.1)	−0.21	
Time on dialysis, *n* (%)				0.28				0.79
Q1 (2–290 days)	72 (27)	24 (21)	−0.16		17 (19)	20 (22)	0.08	
Q2 (290–775 days)	66 (25)	29 (25)	0.00		19 (21)	23 (26)	0.10	
Q3 (775–1846 days)	60 (22)	36 (31)	0.18		29 (33)	26 (29)	−0.07	
Q4 (1846–4811 days)	69 (26)	28 (24)	−0.04		24 (27)	20 (22)	−0.11	
Chronic heart failure, *n* (%)	187 (70)	74 (63)	−0.14	0.19	56 (63)	62 (70)	0.15	0.43
Atrial fibrillation, *n* (%)	210 (79)	75 (64)	−0.30	0.004	60 (67)	61 (69)	0.02	>0.99
Permanent	62 (23)	22 (19)	−0.11		22 (25)	21 (24)	−0.03	
Temporary	18 (7)	9 (8)	0.04		6 (7)	6 (7)	0	
Unknown	130 (49)	44 (37)	−0.28		32 (36)	34 (38)	0.03	
Atrial flutter, *n* (%)	17 (6.4)	10 (8.5)	0.08	0.52	8 (9)	8 (9.0)	0.00	> 0.99
Complete atrioventricular block, *n* (%)	129 (48)	59 (50)	0.05	0.74	45 (51)	47 (53)	0.04	0.88
Other block, *n* (%)	61 (23)	34 (29)	0.14	0.20	24 (27)	26 (29)	0.05	0.87
Sinus dysfunction, *n* (%)	96 (36)	31 (26)	−0.21	0.078	27 (30)	28 (31)	0.02	>0.99
Ischaemic heart disease, *n* (%)	135 (51)	67 (57)	0.14	0.27	49 (55)	51 (57)	0.05	0.88
Ventricular arrhythmia, *n* (%)	15 (5.6)	4 (3.4)	−0.12	0.45	4 (4.5)	3 (3.4)	0.04	>0.99
Supraventricular arrhythmia, *n* (%)	35 (13)	9 (7.7)	−0.20	0.16	8 (9)	9 (10)	−0.06	>0.99
Pulmonary hypertension, *n* (%)	83 (31)	33 (28)	−0.06	0.63	20 (22)	29 (33)	0.22	0.18
Tricuspid valve disease, *n* (%)	38 (14)	16 (14)	−0.02	> 0.99	11 (12)	13 (15)	0.06	0.83
Aortic valve disease	21 (8)	16 (14)	0.17	0.07	10 (11)	13 (15)	0.10	0.51
Mitral valve disease	42 (16)	24 (21)	0.12	0.25	13 (15)	21 (24)	0.21	0.13
Pulmonary valve disease	1 (0)	0 (0)	−0.07	0.51	0 (0)	0 (0)	0	–
Aortic aneurysm, *n* (%)	23 (8.8)	7 (6)	−0.11	0.42	7 (7.9)	7 (7.9)	0.00	>0.99
Peripheric arteritis, *n* (%)	141 (53)	56 (48)	−0.10	0.38	52 (58)	45 (51)	−0.16	0.37
Stroke, *n* (%)	48 (18)	23 (20)	0.04	0.78	19 (21)	19 (21)	0.00	>0.99
Diabetes, *n* (%)	185 (69)	84 (72)	0.06	0.72	66 (74)	68 (76)	0.05	0.86
History of cancer (any type), *n* (%)	80 (30)	39 (33)	0.07	0.55	33 (37)	32 (36)	−0.02	>0.99
Number of pacemakers implanted by the hospital (2009–2020), *n* (%)				<0.001				0.98
Q1 (2–28)	93 (35)	8 (6.8)	−1.11		8 (9)	8 (9)	0.00	
Q2 (28–48)	84 (31)	14 (12)	−0.60		16 (18)	14 (16)	−0.06	
Q3 (48–78)	53 (20)	36 (31)	0.24		31 (35)	31 (35)	0.00	
Q4 (78–362)	37 (14)	59 (50)	0.73		34 (38)	36 (40)	0.05	

Quantitative variables are presented as means and SDs, followed by medians and IQRs. Differences were assessed using the Wilcoxon signed-rank test and evaluated using SMDs. Qualitative variables are described as number of subjects and frequencies (%). Differences were tested using the Student’s *t*-test and assessed using SPDs.

Q, quartiles.

To account for cluster effects related to the institution that performed pacemaker implantation, we included the number of pacemaker implantations performed by each centre in the last decade as a predictive variable in the PS.

Survival was compared between groups using a univariate Cox proportional hazards model in the matched sample, and survival curves were performed using the Kaplan–Meier method. A sensitivity analysis was performed with an adjustment with age and cluster effects.^27,28^ In all cases, individual risk was considered as random effect. In-hospital mortality was compared using a Fisher test.

Acute complications (within the first 90 days) and long-term complications (including those occurring within the first 90 days) were reported in both the unmatched and matched samples, with the number and frequency of events. In a time-to-event analysis, acute and long-term complications, as well as first intervention on vascular access, were compared using a Fine and Gray model, which allowed us to account for competing risks of death. Individual risk was considered as random effect. If there were no events in a particular complication category for one group, we used a log-rank test. When the sample size was small, we conducted a descriptive analysis instead. We then compared the rate of surgical interventions on vascular access using Poisson’s regression. Finally, we compared the duration of hospitalization using a Student’s *t*-test and provided a comparison to a historical cohort with PS matching.

Significance threshold was set at *α* = 0.05, and all *P*-values were two-tailed. Statistical analyses were conducted using R version 4.2.2.

## Ethical approval

This French Rein registry got the approval of the National Committee for Civil Liberties and Data Protection, CNIL (approval no. 903, 188).

## Results

### Cohort formation and baseline characteristics

Out of 168 273 ESKD patients aged ≥ 18 years recorded in the REIN database between 2009 and 2020, 118 666 were successfully matched with the SNDS database, resulting in a matching rate of 70.5%. After excluding patients based on the exclusion criteria, there were 267 patients in the single-chamber transvenous pacemaker group and 117 patients in the leadless pacemaker group (*Figure 1*). Then, we conducted matching based on PSs, resulting in a sample of 89 patients in each group, totalling 178 patients for analysis. Balance between baseline characteristics with PS matching was satisfactory (*Table 1*, see Supplementary material online, *Figure S1*), as well as with IPTW (see Supplementary material online, *Figure S2*). Information on device model was available in 21 of the 89 patients in leadless pacemaker group, and all of these 21 patients were implanted with a Medtronic Micra® pacemaker.

### Survival

**Figure 2:**
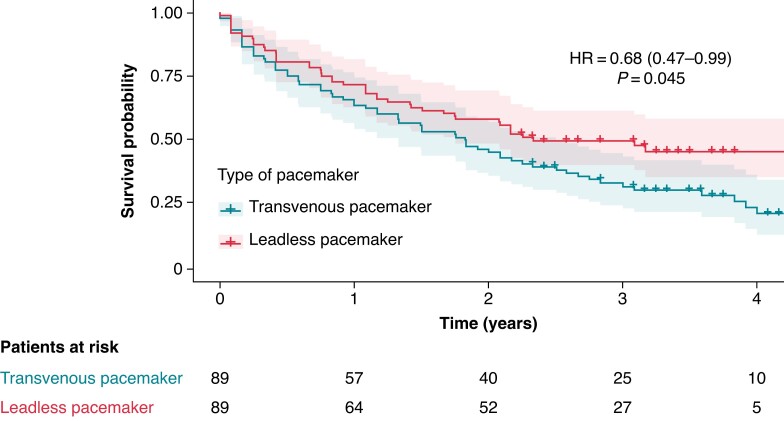
Comparison of survival after leadless and transvenous pacemaker implantation in the propensity score-matched sample. The hazard ratio presented here is derived from a univariate Cox regression model conducted after propensity score matching. Individual risk is accounted for by a random effect at the individual level. HR, hazard ratio.

*Figure 2* displays the results of the survival analysis. Median duration of follow-up was 22 months [interquartile range (IQR) 7–37 months] for transvenous pacemaker and 27 months (IQR 10–38 months) for leadless pacemaker. There was no significant difference of follow-up duration between groups (*P* = 0.5). There was a significant lower all-cause mortality rate in leadless pacemaker compared to transvenous [hazard ratio (HR) = 0.68, 95% confidence interval (95% CI 0.47–0.99)], confirmed in sensitivity analysis adjusted on age and cluster effects after PS matching (HR = 0.67, 95% CI 0.46–0.99) (see Supplementary material online, *Table S2*). Sensitivity analyses applying the IPTW with PS were of the same magnitude (HR = 0.66, 95% CI 0.41–1.04, after adjustment on age and centre effect: HR = 0.65, 95% CI 0.42–1.01) (see Supplementary material online, *Table S2*). In-hospital mortality was 1.1% in the leadless pacemaker group and 7.9% in the transvenous group, with no statistically significant difference between the groups [odds ratio (OR) 0.13, 95% CI 0.003–1.08, *P* = 0.064].

### Acute complications

For data concerning complications, median follow-up duration was 13 months (IQR 5–21 months) in transvenous pacemaker and 8 months (IQR 3–14 months) in leadless pacemaker, with a significant difference between groups (*P* = 0.004). The results of comparative analyses regarding acute complications are presented in *Table 2*. There was no difference in the occurrence of cardiac arrests within 90 days following pacemaker implantation. Only one case of haemopericardium occurred in the leadless pacemaker group (1.1%). No cases of pneumothorax or haemothorax were observed in either group. There was also no statistical difference observed regarding the number of haemorrhages or complications at the site of access during pacemaker implantation. Concerning DVT/PE, there were three cases in the transvenous pacemaker group and four cases in the leadless pacemaker group. There was no significant difference between groups (HR 1.32, 95% CI 0.30–5.86). There were significantly fewer endocarditis or device-related infections with leadless pacemakers (0 cases vs. 4 cases in the transvenous group, HR 0.37, 95% CI 0.14–0.98). A number of acute complications occurring in the whole cohort are reported in Supplementary material (see Supplementary material online, *Table S3*). The average length of hospital stay in leadless pacemaker group was shorter than for transvenous pacemaker group (6.79 vs. 11.17 days, mean difference: −4.38 days, 95% CI −8.18 to −0.58 days) and shorter to the average length of stay in haemodialysis patient implanted with a transvenous pacemaker between 2009 and 2017 (11.62 days, mean difference: −4.83 days, 95% CI −8.44 to −1.22 days). The average time from hospitalization to pacemaker implantation was 3.23 days in leadless pacemaker group and 4.80 days in transvenous pacemaker group, showing no significant difference (mean difference: −1.65, 95% CI −3.48 to 0.31).

**Table 2:** Acute and long-term complications and interventions on vascular access after leadless compared to transvenous pacemaker implantation in the propensity score-matched sample

Adverse events	Transvenous pacemaker, *n* = 89	Leadless pacemaker, *n* = 89	HR/RR	95% CI	*P*-value
Acute (<90 days)					
Cardiac arrest, *n* (%)	7 (7.9)	3 (3.4)	0.43	(0.11–1.67)	0.22
Haemopericardium, *n*(%)	0 (0)	1 (1.1)			
Pneumothorax/haemothorax, *n* (%)	0 (0)	0 (0)			
Haemorrhage, *n* (%)	6 (6.7)	4 (4.5)	0.66	(0.19–2.35)	0.52
Complication at the site of access (pacemaker implantation), *n* (%)	2 (2.2)	8 (9)	4.25	(0.88–20.39)	0.070
DVT/PE, *n* (%)	3 (3.4)	4 (4.5)	1.32	(0.30–5.86)	0.71
Endocarditis/device-related infection, *n* (%)	4 (4.5)	0 (0)	0.37	(0.14–0.98)	0.045
Long term					
Endocarditis/device-related infection, *n* (%)	8 (9)	0 (0)	0.43	(0.21–0.86)	0.0093
DVT/PE, *n* (%)	11 (13)	9 (7.9)	0.96	(0.41–2.20)	0.92
Intervention on haemodialysis vascular access					
At least one, *n* (%)	30 (33)	17 (19)	0.62	(0.35–1.08)	0.090
Total number of interventions (*n*)	75	27			
Time of exposure (py)	91.66	67.56			
Annual rate (*n*/10 py, RR)	8.18	3.99	0.49	(0.31–0.75)	0.0014
Type of intervention (*n*/10 py, RR)					
First AVF creation	0.76	0.30	0.39	(0.06–1.61)	0.23
Ulterior AVF creation	0.76	0.44	0.58	(0.13–2.08)	0.43
Thrombectomy/angioplasty	6.33	2.96	0.47	(0.27–0.76)	0.0034
AVF closure/aneurysm resection	0.33	0.30	0.90	(0.12–5.46)	0.91

Survival prior to complication occurrence was assessed using a Fine and Gray model to consider the competing risk of death. Hazard ratios are presented for time-to-event analysis. Surgical rates were compared using Poisson regression, and relative risks are provided for reference. Statistical comparison was not performed when sample sizes were too small.

95% CI, 95% confidence interval; py, person-years; DVT/PE, deep vein thrombosis/pulmonary embolism; AVF, arteriovenous fistula.

### Long-term complications

Results concerning the analysis of long-term complications are also presented in *Table 2*. A number of long-term complications occurring in the whole cohort are reported in Supplementary material (see Supplementary material online, *Table S3*). When focused on the matched sample, endocarditis or device-related infections were observed in eight patients in the transvenous group, and none occurred in the leadless pacemaker group, which was a significantly lower rate in the leadless group (HR 0.43, 95% CI 0.21–0.86). The median age of patients with device-related infection was 75 years. The median time between pacemaker implantation and diagnosis of infection was 76 days. Among the eight infected patients, death occurred in seven, with a median time between diagnosis and death of 444 days. Individual information of each patient diagnosed with device-related infection is available in Supplementary material online, *Appendix S1* (see Supplementary material online, *Table S4*). There was no difference in the occurrence of DVT/PE between both groups during all follow-up.

### Complications related to vascular access for haemodialysis

We investigated interventions on vascular access for haemodialysis, comparing time before the first intervention and the rates of interventions between transvenous and leadless pacemakers. Time before the first intervention was not significantly different between groups (HR 0.62, 95% CI 0.35–1.08). The rate of surgery related to vascular access was significantly lower in the leadless group [relative risk (RR) 0.49, 95% CI 0.31–0.75]. Comparison of categories of surgery showed that this difference was mainly explained by a lower number of thrombectomy or angioplasty on AVF in the leadless group (*Table 2*).

## Discussion

This real-world study compared the survival and adverse events related to leadless, vs. single-chamber transvenous, pacemakers specifically in haemodialysis patients. Several important results were highlighted in our study. First, survival, after accounting for confounding factors, was higher following the implantation of a leadless pacemaker compared to a single-chamber transvenous pacemaker in haemodialysis patients. This increase in survival was consistent in sensitivity analyses.

In-hospital mortality appeared to be lower with leadless pacemakers, though the difference between groups was not statistically significant (OR 0.13, *P* = 0.06). Alhuarrat *et al.*^14^ found in an American, mostly non-dialysis dependent, population a higher likelihood of in-hospital mortality associated with leadless pacemakers compared to transvenous pacemakers, possibly due to higher comorbidities in the leadless pacemaker group. Our study population is significantly different from the study by Alhuarrat *et al.*, since we only included haemodialysis, highly comorbid patients, in a French setting.

When focusing on acute adverse events related to pacemaker implantation, we did not find any significant difference between groups in the occurrence of cardiac arrests, haemorrhage, complications at the site of access for pacemaker implantation, or in thrombosis/PE. Only one haemopericardium occurred in the leadless pacemaker group. It is a known but infrequent complication related to leadless pacemaker implantation.^29^ The low rate of acute complications (haemothorax, pneumothorax) following pacemaker implantation in our study suggests a high level of expertise from the physicians who performed these procedures, for both types of devices.

The duration of hospitalization following leadless pacemaker implantation was shorter than that following transvenous pacemaker implantation, even in haemodialysis patients implanted with a single-chamber transvenous pacemaker before 2017. It was previously reported in a non-haemodialysis population that the duration of initial hospitalization following leadless and transvenous pacemakers implantation was similar,^14^ but no data have been published, to our knowledge, in haemodialysis patients. In addition, PS matching allowed to account for baseline characteristics. Hence, our findings support a shorter hospital stay in haemodialysis patients receiving a leadless pacemaker, likely due to favourable outcomes following pacemaker implantation rather than a selection bias prior to implantation.

Analyses of acute and long-term complications showed a significantly higher risk of endocarditis or device-related infections in the transvenous group compared to the leadless group. Dialysis patients represent a population at high risk for device-related infections due to frequent exposure to bacteraemia,^5,30^ which may account for the elevated incidence of device-related infections. Other known risk factors for this complication include male sex, diabetes, and immunosuppressive therapy.^28,29^ Additionally, factors related to the device itself, such as the need for reintervention or lead replacement, pocket haematoma, and abandoned leads, contribute to the presence of device-related infections specifically in transvenous pacemakers.^30^ No device-related infections occurred in leadless pacemaker group. Consistent with our findings, other studies have reported a very low risk of device-related infections associated with leadless pacemakers.^31,32^ Device-related infections are associated with a poor prognosis in haemodialysis patient.^7^ While time to first intervention on vascular access for haemodialysis was not significantly different between both groups, the rate of intervention was significantly higher in the transvenous pacemaker group. This was mainly explained by the number of thrombectomies or angioplasties of the AVF. The rate of AVF creation was no different between groups, suggesting that they did not impact AVF creation.

Only one previous study compared leadless to transvenous pacemakers in ESKD patients, to our knowledge, and did not demonstrate a significant benefit in survival or reduction of adverse events in this population, despite having a large sample size of ESKD patients.^19^ That study included all ESKD patients (CKD Class 4 or 5, haemodialysis, peritoneal dialysis, and kidney recipients) and was not limited to haemodialysis patients specifically. Our study focused solely on haemodialysis patients, in period of time where both devices were authorized and could be implanted according to the cardiology team preference, leading to a higher number of transvenous pacemakers than in leadless pacemakers. This situation is optimal for assuring covariate balance with PS matching.^26^ Despite a relatively small number of patients in our study, we were able to find a significant difference in survival. These results differ with those obtained in the study by Boveda *et al.*^19^ This difference could partially be attributed to differences in definition of ESKD populations and variations in practices between France and the USA. Additionally, the duration of follow-up in our study is longer, with a median follow-up of 2 years and extending up to 5 years, possibly allowing us to capture more long-term complication events.

A second substantial benefit highlighted was the reduction in the number of interventions required for vascular access. The Kidney Disease Outcomes Quality Initiative guidelines recommend native AVF as the primary vascular access for haemodialysis patients due to higher quality of life and a lower rate of complications.^33^ Ensuring the presence of a high-quality vascular access for haemodialysis is a priority in ESKD patients. Our study found that leadless pacemaker implantation was associated with a reduction in the need for thrombectomy/angioplasty on AVF, likely attributable to a decrease in the occurrence of central venous stenosis. Central venous stenosis is frequently observed following transvenous pacemaker implantation, which can be explained by surgical access through a central upper arm vein.^8^

### Limitations

Our study has several limitations. First, although we utilized real-world data from large national registries, it remains a retrospective observational study, which does not allow for the assertion of causal relationships. Since patients were not randomized, an indication bias may have been introduced. To minimize this bias, we employed rigorous PS matching to enhance the comparability between groups, but this cannot fully compensate for the absence of randomization. No randomized controlled trial (RCT) has yet been completed or is ongoing to compare the efficacy and safety of leadless vs. transvenous pacemakers in an haemodialysis population. Leadless pacemakers are already widely used in routine clinical practice, and while RCTs are considered the gold standard for determining efficacy, they are expensive and difficult to implement, particularly for devices already integrated into everyday care. In this context, registry-based observational studies offer a practical and valuable approach to comparing these devices and evaluating outcomes in a real-world setting.

Second, 71% of the patients in the REIN database were successfully matched with the SNDS database. It is likely that the unmatched patients are predominantly from earlier data periods, as our study focused on prevalent dialysis patients who could not be identified in the SNDS if they had started dialysis before 2006 (see Supplementary material online, *Methods S1*). Moreover, the SNDS coverage was only partial before 2009, becoming almost complete after that year, which increased the likelihood of finding matches in the SNDS. For instance, the reported matching rate between REIN and SNDS was of 87% in patients who began dialysis after 2009.^20^ Since our study concerned prevalent haemodialysis patients between 2017 and 2020, it is likely that the matching rate is significantly higher than 70%, although it cannot be determined with complete accuracy.

Third, another limitation is the relatively small sample size of leadless pacemaker recipients in our study. Only 117 haemodialysis patients received a leadless pacemaker during the study period, resulting in 89 participants in this group after matching. To ensure balanced groups, several patients had to be excluded. Although we accounted for this through a sensitivity analysis using the IPTW method, it may impact the generalizability of our results.

Fourth, while we found significantly less infectious events in the matched leadless pacemaker group than in the transvenous group, we had no specific information on these infectious events and we also report three infectious events in the unmatched leadless group. Hence, the reduction of infectious events with leadless pacemakers in haemodialysis patients needs to be confirmed in additional studies comparing both devices.

Fifth, we did not have data on left ventricular ejection fraction in patients with chronic heart failure, a known risk factor for mortality. However, it is unlikely that this parameter explains the survival differences observed after PS matching. Additionally, we lacked information on whether the pacemaker was implanted on the same side as an existing AVF. It is well established that pacemaker implantation on the same side as an AVF can increase the risk of stenosis or thrombosis. International guidelines recommend placing pacemakers on the opposite side of an AVF to minimize these risks, and it is likely that physicians adhered to these guidelines in clinical practice.

Finally, we were unable to apply the same censoring date for both survival and complication analyses due to data availability in the database. We chose to retain all available information for the survival analysis to ensure comprehensive data utilization, and to censor the death date earlier solely for the complication analysis. The latter enabled us to effectively account for the competing risk of death in the complication comparison, utilizing a robust statistical model. The duration of follow-up significantly differs for complications between groups, but the time-to-event analysis allowed for accounting for this difference in the comparison between groups.

## Conclusions

Our observational study on patients in haemodialysis found an interesting association between implant of a leadless pacemakers and better survival as compared to conventional transvenous pacing. The beneficial effect on survival in this high-risk population deserves confirmation by randomized trials, but could be potentially attributed to a reduction in occurrence of endocarditis or device-related infections that are associated with a high mortality rate in haemodialysis patients.^5,6^ These findings are of particular interest given the exponential development of leadless pacing in the near future, especially the availability of dual-chamber leadless pacemaker.

## Supplementary Material

euae257_Supplementary_Data

## Data Availability

Data were provided by the Agence de la biomédecine (ABM) and will not be made available to other researchers without specific authorization.
