# Supplementary material for: Improved outcomes with leadless vs. single-chamber transvenous pacemaker in haemodialysis patients
Source: Europace. 2024 Oct 1;26(11):euae257. doi: 10.1093/europace/euae257 (PMC11542626; doi:10.1093/europace/euae257)
Supplement: euae257_Supplementary_Data [file euae257_supplementary_data.zip › appendix_R1_europace.docx]

**Supplementary files**

**Methods:**

**Data collection from Système National des Données de Santé (SNDS)**

Table S1 displays the Common Classification of Medical Acts (CCAM) and International Classification of Diseases - 10 (ICD-10) codes utilized for identifying comorbidities and complications. The SNDS database contains data collected during almost every hospitalization in France. Information about diagnoses is provided in two main ways. The "DGN_PAL" column identifies the primary diagnosis associated with the hospitalization. Additionally, there are several columns identifying associated diagnoses related to the hospitalization. Associated diagnoses may pertain to the patient's pre-existing comorbidities or to acute issues that developed during the hospitalization. For comorbidities at inclusion, patients were considered to have a comorbidity if it was documented as "Yes" in either the Réseau Epidémiologie et Information National (REIN) database or the SNDS database before the date of pacemaker implantation. Codes identified as main or secondary diagnoses were taken into consideration for comorbidities.

For complications, only data from the SNDS database were considered. We utilized every primary diagnosis to identify complications, and secondary diagnoses were used only if no identical code was found in the patient's history. For instance, if a patient had no prior history of thrombosis in the SNDS database but received a thrombosis-related code after pacemaker implantation, we included this code as indicating a thrombosis complication. However, if a patient had any code related to thrombosis (either as a primary or secondary diagnosis) before pacemaker implantation and then received a thrombosis-related code as a secondary diagnosis after implantation, we did not include this code.

**Matching process between REIN and SNDS database**

The REIN registry manually collects data on all end-stage kidney disease (ESKD) patients at the initiation of their first renal replacement therapy (RRT) across France and has been recording data on ESKD patients since 2002. The SNDS database contains pseudonymized medico-administrative data used for reimbursement and hospital activity tracking, covering 99% of the current population. The SNDS has recorded data since 2006, with coverage reaching 99% of the population by 2009. The matching process began by identifying the first renal replacement therapy in both databases, followed by matching based on variables such as age, sex, and others. Our baseline population consisted of prevalent hemodialysis patients between 2009 and 2020, but hemodialysis could have started before 2006. As a result, all patients who began hemodialysis before 2006 could not be matched in the SNDS, which explains the matching rate of 70.5%.

An evaluation of the matching process by Raffay et al. (1) shows that the matching rate for incident hemodialysis patients after 2009 reached 87%. Given that our final cohort includes prevalent hemodialysis patients between 2017 and 2020, it is highly likely that the matching rate for this cohort is significantly higher than 70%, although we do not have precise data available. We provided in the manuscript a flowchart that represents the process used to construct our final cohort.

**Covariates balance**

In our sensitivity analysis, we reproduced the primary analysis using inverse probability of treatment weighting (IPTW) instead of propensity score (PS) matching to assess covariates balance. IPTW allowed us to retain all 384 subjects in the analysis. It consists of applying a weight to all eligible participants in the analysis based on the PS. Individuals who received leadless pacemakers were weighted as 1/PS, and individuals who received transvenous pacemakers were weighted as 1/(1-PS). The decision to use PS matching instead of IPTW as the primary analysis was made after measuring the effective sample size with both methods and then calculating the effective power (2). When applying weighting techniques such as Inverse Probability of Treatment Weighting (IPTW) in statistical analyses, the effective sample size may undergo alteration. IPTW is employed to mitigate potential biases arising from imbalances in the characteristics of compared groups. However, this weighting approach can also influence the contribution of individual observations to the analysis, thereby impacting the effective sample size. Mathematically, the effective sample size (nEff) after applying IPTW can be expressed as:

$$nEff= {(\sum wi)}^{2}{}/{{\sum(wi}^{2})}$$

Where wi represents the weight assigned to each observation.

We need to consider the effective sample size when calculating power to assess the impact of weighting on power. The effective sample size with matching was 89 patients per group, totaling 178 patients, resulting in 44% power for a two-sided Cox model. With IPTW, the effective sample size was 34.86 patients in the leadless pacemaker group and 206.46 patients in the transvenous pacemaker group, resulting in 31% power for a two-sided Cox model. Therefore, we opted for propensity score matching as the primary analysis in our study. The decision to conduct 1:1 ratio matching instead of 2:1 was driven by the aim to achieve the optimal balance between maximizing the effective sample size and attaining the most favorable covariate balance. Opting for a 2:1 ratio resulted in an effective sample size of 114.8 in the transvenous group and 89 in the treated group, yielding a 48% power increase, representing a gain of 4%, but at the expense of a greater disparity between the groups.

Figure S1 and Figure S2 depict covariate balance before and after propensity score matching and weighting, respectively.

**Constitution of a historical cohort**

After creating a propensity score-matched cohort of 89 patients who received a leadless pacemaker, we constructed a comparative historical cohort of 89 patients who received a transvenous pacemaker between 2009 and 2017, prior to the first hemodialysis patient in our cohort receiving a leadless pacemaker. Matching was done using the nearest propensity score (PS) in the historical cohort, which was built using the same variables. We used this cohort to compare the duration of hospital stay (specifically the stay during which the pacemaker was implanted) and to assess survival through supplemental analysis using Cox regression on the matched sample (leadless and historical cohorts).

**Results:**

**Sensitivity analyses**

Table S2 presents the results of the comparison of overall survival with sensitivity analyses. Propensity score matching with adjustment for cluster effects revealed a hazard ratio (HR) of 0.67 (95 percent Confidence Interval (CI95): 0.47-0.99), indicating a significant survival benefit with leadless pacemakers. Sensitivity analyses applying the inverse probability of treatment weighting (IPTW) with PS were of the same magnitude (HR = 0.66, 95% CI: 0.41 – 1.04, after adjustment on center effect: HR = 0.65, 95% CI: 0.42 – 1.01)

**Comparison of survival between leadless pacemaker group and historical cohorte of transvenous pacemaker**

Survival was statistically higher in hemodialysis patients implanted with a leadless pacemaker than in the historical cohort implanted with a transvenous pacemaker before 2017 (HR : 0.53, 95% CI: -1.00 - -0.27, p < 0.001).

**Description of patients diagnosed with device-related infection in the transvenous pacemaker group**

**Table S3** present acute and long term complications in the whole cohort. Regarding endocarditis or device related infection in the overall sample before propensity score matching, 17 events occurred in the transvenous pacemaker group and 3 events occurred in the leadless. We do not provide a statistical comparison since these groups are not comparable at baseline. **Table S4** presents characteristics and outcomes of 8 patients diagnosed with device related infection in the transvenous pacemaker group in the macthed sample. Eight patients implanted with a single-chamber transvenous pacemaker developed a device-related infection during the follow-up. Among them, five (65.2%) were male. The median age was 75 years [Interquartile Range (IQR) 67 – 84], and the median body mass index (BMI) was 25.5 kg/m2 [IQR 20.5 – 31.8]. Seven patients (87.5%) had diabetes. Five patients had an arteriovenous fistula as vascular access for hemodialysis at the time of pacemaker implantation. The median time between pacemaker implantation and diagnosis of device-related infection was 76 days [IQR 5 – 166]. Death occurred in seven patients, with a median time between diagnosis and death of 444 days [IQR 33 – 715].

**Supplemental bibliography:**

1. Raffray M, Bayat S, Lassalle M, Couchoud C. Linking disease registries and nationwide healthcare administrative databases: the French renal epidemiology and information network (REIN) insight. BMC Nephrol. déc 2020;21(1):25.

2. Austin PC. Informing power and sample size calculations when using inverse probability of treatment weighting using the propensity score. Stat Med. 30 nov 2021;40(27):6150‑63.

**Tables and figures legends**

**Table S1. ICD-10 and CCAM codes associated with comorbidities, complications, pacemaker implantation or control, and vascular access surgeries.**

**Figure S1. Covariates balance before and after propensity score matching.** The solid line represents a standardized mean or proportion difference (SMD/SPD) of 0, indicating perfect balance for the variable of interest between the two groups (single-chamber transvenous pacemaker and leadless pacemaker). Dashed lines correspond to absolute threshold values of 0.15. A standardized difference less than 0.15 indicates balance between the two groups for the variable. Multinomial qualitative variables were dichotomized for each level of the variable to control standardized differences at each level. BMI = body mass index.

**Figure S2. Covariates balance before et after weighting with IPTW method**. The solid line represents a standardized mean or proportion difference (SMD/SPD) of 0, indicating perfect balance for the variable of interest between the two groups (single-chamber transvenous pacemaker and leadless pacemaker). Dashed lines correspond to absolute threshold values of 0.15. A standardized difference less than 0.15 indicates balance between the two groups for the variable. Multinomial qualitative variables were dichotomized for each level of the variable to control standardized differences at each level. BMI = body mass index.

**Table S2. Survival analysis of hemodialysis patient with leadless pacemaker compared to transvenous pacemaker after matching with propensity score (with and without adjustment on center effects) and after IPTW (with and without adjustment on center effects).** Hazard ratio for risk of death. 95% CI = 95% Confidence interval; IPTW = Inverse propensity of treatment weighting

**Table S3. Description of acute and long-term complications and interventions on vascular access in the whole wohort.** Statistical comparison was not performed as population were not comparable as baseline. py=person-years, DVT/PE= deep vein thrombosis/pulmonary embolism, AVF = arteriovenous fistula.

**Table S4. Description of each patient with device related infection after single-chamber transvenous pacemaker implantation**. DVI= Device Related Infection. Q= Quartiles.
