# Supplementary material for: Improved outcomes with leadless vs. single-chamber transvenous pacemaker in haemodialysis patients
Source: Europace. 2024 Oct 1;26(11):euae257. doi: 10.1093/europace/euae257 (PMC11542626; doi:10.1093/europace/euae257)
Supplement: euae257_Supplementary_Data [file euae257_supplementary_data.zip › Table_S1_codes_SNDS_v4_AP.docx]

| Pacemakers | Common Classification of Medical Acts (CCAM) codes |
| --- | --- |
| Single-chamber transvenous pacemaker | "DELF007" |
| Leadless pacemaker | "DELF223" |
| Other pacemakers or defibrillator | "DELA001", "DELA003", "DELF001", "DELF005", "DELF010", "DELF012", "DELF015", "DELF901", "DELF902", "DELF903", "DELF904", "DELF905", |
| Event related to pacemakers or defibrillators | "DEEA001", "DEEF001", "DEGF001", "DEGF002", "DEGF003", "DEGF004",  "DEGF005", "DEGF006", "DELF017", "DELF019", "DEGA003", "DEGF207",  "DEKA001", "DELA006", "DEMP002" |
|  |  |
| BASELINE COMORBIDITIES | International Classification of Disease 10th edition (ICD-10) codes |
| Heart failure | "I09.81", "I11.0", "I13.0", "I13.2", "I50", "I500", "I501", "I509", "I5000", "I5001", "I5002", "I5009", "I5010","I5011", "I5012", "I5019" |
| Atrial fibrillation | "I48","I480","I481","I482","I489" |
| Atrial flutter | "I483","I484","I4892" |
| Complete atrioventricular block | "I441","I442","I443" |
| Others block | "I444","I445","I446","I447","I45","I450","I451","I452","I453","I454","I455", "I458","I459" |
| Sinusal dysfunction | "I495","I498","R001" |
| Ischemic heart disease | "I200","I201","I208","I209","I210", "I2100", "I21000", "I2108", "I211", "I2110", "I21100", "I2118", "I212", "I2120", "I21200", "I2128", "I213", "I2130", "I21300", "I2138", "I214", "I2140", "I21400", "I2148", "I219", "I2190", "I21900", "I2198", "I220", "I2200", "I22000", "I2208", "I221", "I2210", "I22100", "I2218","I228","I2280","I22800", "I2288", "I229", "I2290", "I22900", "I2298", "I24","I248","I249","I25", "I251", "I255", "I258", "I259", "Z867", "Z8671" |
| Ventricular arrhythmia | "I472","I490" |
| Supraventricular arrhythmia | "I471","I498" |
| Pulmonary hypertension | "I27","I270","I271","I272","I278","J44","J440","J441","J448","J449" |
| Tricuspid valve disease | "I07","I070","I071","I078","I079","I080","I081","I082","I083","I36","I360",  "I361","I362","I368","I369","Q224","Q228","Z952" |
| Aortic aneurysm | "I71","I710","I711","I712","I713","I714","I715","I716","I718","I719" |
| Peripheric arteritis | "I702","I7020","I7021","I708","I7080","I7081","I743","I744","I745","I771" |
| Stroke | "G45","G450","G458","G451","G459","G46","G463","G464",  "G468","I60","I613","I63","I630","I631","I632","I633",  "I634","I635","I636","I638","I639","I64","I69","I693",  "I694","I698", "I694","Y658","Z866","Z8660" |
| Diabetes | "E10","E100","E101","E102","E103","E104","E105","E106","E107","E108", "E109", "E11","E110","E1100","E111","E112","E113","E114","E115","E116","E117",  "E1110", "E1130", "E1140", "E1170", "E118", "E1118", "E1128", "E1138", "E1148", "E1150", "E1158", "E1168", "E1178", "E1188", "E119", "E1190", "E14", "E140", "E141", "E143", "E144", "E145", "E146","E147","E148","E149","E126", |
| Cancer (all type) | "C00", "C000", "C001", "C002", "C003", "C004", "C006", "C008", "C009", "C01", "C02", "C020", "C021", "C022", "C023", "C024", "C028", "C029", "C03", "C030", "C031", "C039", "C04", "C040", "C041", "C048", "C049", "C05", "C050", "C051", "C058", "C059", "C06", "C060", "C061", "C062", "C068", "C069", "C07", "C08", "C080", "C081", "C088", "C089", "C09", "C090", "C091", "C098", "C099", "C10", "C100", "C101", "C102", "C103", "C104", "C108", "C109", "C11", "C110", "C111", "C112", "C113", "C118", "C119", "C12", "C13", "C130", "C131", "C132", "C138", "C139", "C14", "C140", "C142", "C148", "C15", "C150", "C151", "C152", "C153", "C154", "C155", "C158", "C159", "C16", "C160", "C161", "C162", "C163", "C164",  "C165", "C166", "C168", "C169", "C17", "C170", "C171", "C172", "C173", "C178", "C179", "C18", "C180", "C181", "C182", "C183", "C184", "C185", "C186", "C187", "C188", "C189", "C19", "C20", "C21", "C210", "C211", "C212", C218", "C22", "C220", "C221", "C222", "C223", "C224", "C227", "C229", "C23", "C24", "C240", "C241", "C248", "C249", "C25", "C250", "C251", "C252", "C253", "C254", "C257", "C258", "C259", "C26", "C260", "C261", "C268", "C269", "C30", "C300", "C301", "C31", "C310", "C311", "C312", "C313", "C318", "C319", "C32", "C320", "C321", "C322", "C323", "C328", "C329", "C33", "C34", "C340", "C341", "C342", "C343", "C348", "C349", "C37", "C38", "C380", "C381", "C382", "C383", "C384", "C388", "C39", "C390", "C398", "C399", "C40", "C400", "C401", "C402", "C403", "C408", "C409", "C41", "C410", "C411", "C412", "C413", "C414", "C418", "C419", "C43", "C430", "C431", "C432", "C433", "C434", "C435", "C436", "C437", "C438", "C439", "C44", "C440", "C441", "C442", "C443", "C444", "C445", "C446", "C447", "C448", "C449", "C45", "C450", "C451", "C452", "C458", "C459", "C46", "C460", "C461", "C462", "C463", "C467", "C468", "C469", "C47", "C470", "C471", "C472", "C473", "C474", "C475", "C476", "C477", "C478", "C479", "C48", "C480", "C481", "C482", "C488", "C49", "C490", "C491", "C492", "C493", "C494", "C495", "C496", "C498", "C499", "C50", "C500", "C501", "C502", "C503", "C504", "C505", "C506", "C508", "C509", "C51", "C52", "C53", "C54", "C55", "C56", "C57", "C58", "C60", "C61", "C62", "C63", "C64", "C65", "C66", "C67", "C68", "C69", "C70", "C71", "C72", "C73", "C74", "C740", "C741", "C749", "C75", "C750", "C751", "C752", "C753", "C754", "C757", "C758", "C759", "C76", "C760", "C761", "C762", "C763", "C764", "C765", "C767", "C768", "C77", "C770", "C771", "C772", "C773", "C774", "C775", "C778", "C779", "C78", "C780", "C781", "C782", "C783", "C784", "C785", "C786", "C787", "C788", "C79", "C790", "C791", "C792", "C793", "C794", "C795", "C796", "C797", "C798", "C80", "C800", "C81", "C810", "C811", "C812", "C813", "C817", "C819", "C82", "C820", "C821", "C822", "C827", "C829", "C83", "C830", "C831", "C832", "C833", "C834", "C835", "C836", "C837", "C838", "C839", "C84", "C840", "C841", "C842", "C843", "C844", "C845", "C85", "C850", "C851", "C857", "C859", "C88", "C880", "C881", "C882", "C883", "C887", "C889", "C90", "C900", "C901", "C902", "C91","C910", "C911", "C912", "C913", "C914", "C915", "C917", "C919", "C92", "C920", "C921", "C922", "C923", "C924", "C925", "C927", "C929", "C93", "C930", "C931", "C932", "C937", "C939", "C94", "C940", "C941","C942", "C943", "C944", "C945", "C947", "C95", "C950", "C951", "C952", "C957", "C959", "C96", "C960", "C961", "C962", "C963", "C967", "C969", "C97" |
|  |  |
| COMPLICATIONS | ICD-10 codes |
| Cardiac arrest | "I46","I460", "I461", "I469" |
| Hemopericardium | "I312", "S26", "S260", "S2600","S2601", "S268", "S2680", "S2681" ,"S269", "S2690", "S2691" |
| Pneumothorax / Hemothorax | "J942","S27","S270", "S2700", "S2701", "S271", "S2710", "S2711", "S272",  "S2720", "S2721", "S273", "S2730", "S2731","S276", "S2760", "S2761" |
| Hemorraghe | "T810","T792","Y60","Y600","Y605","Y606","Y608","Y609" |
| Complication at the site of vascular access (pacemaker implantation) | "I721", "I724", "I770","I772","I778","I779","T812","T817","S450", "S451", "S452", "S453", "S457", "S458", "S459", "S75","S750", "S751", "S757", "S758", "S759" |
| Deep vein thrombosis | "I80","I800","I801","I802","I803","I808","I809", "I82", "I822", "I823", "I828", "I829" |
| Pulmonary embolism | "I26","I260","I261","I269" |
| Endocarditis or device related infection | "T827","I33","I330","I339" |
|  |  |
| Surgeries | CCAM codes |
| Arteriovenous fistula (AVF) creation | "EZMA001", "EZMA003", "EPCA001" |
| Thrombectomy | "EZPA001","EZJF001","EZJF002","EZPF003" |
| AVF closure | "EZSA001", "EZSA002", "EZSA003", "EZCA003", "EZFA002", "EZFA003" |
| AVF stenosis dilatation | "EZAF002", "EZAF001","EZCA004", "EFAF001","EFAF002","EFPF003","EPPF001" |
| Vein ligation | "EFFA001", "EFSA001" |

**Table S1.** **ICD-10 and CCAM codes associated with comorbidities, complications, pacemaker implantation or control, and vascular access surgeries.**
