# Supplementary material for: Improved outcomes with leadless vs. single-chamber transvenous pacemaker in haemodialysis patients
Source: Europace. 2024 Oct 1;26(11):euae257. doi: 10.1093/europace/euae257 (PMC11542626; doi:10.1093/europace/euae257)
Supplement: euae257_Supplementary_Data [file euae257_supplementary_data.zip › Table_S2_sensitivity_analyses_V3_AP-FG.docx]

| **Survival analysis** | **Hazard-Ratio for death** | **95% CI** | **p-value** |
| --- | --- | --- | --- |
| **Propensity score matching** |  |  |  |
| unadjusted | 0.68 | [0.47-0.99] | 0.045 |
| Adjusted with CE | 0.67 | [0.46-0.99] | 0.042 |
|  |  |  |  |
| **Inverse probability of treatment weighting** |  |  |  |
| unadjusted | 0.66 | [0.41-1.04] | 0.075 |
| Adjusted with CE | 0.65 | [0.42-1.01] | 0.058 |

**Table S2.**  **Survival analysis of hemodialysis patient with leadless pacemaker compared to transvenous pacemaker after matching with propensity score (with and without adjustment on center effects) and after IPTW (with and without adjustment on center effects). Hazard ratio for risk of death.** 95% CI = 95% Confidence interval; CE= center effects.
