# Supplementary material for: Improved outcomes with leadless vs. single-chamber transvenous pacemaker in haemodialysis patients
Source: Europace. 2024 Oct 1;26(11):euae257. doi: 10.1093/europace/euae257 (PMC11542626; doi:10.1093/europace/euae257)
Supplement: euae257_Supplementary_Data [file euae257_supplementary_data.zip › Table_S3_AdverseEvents_WholeCohort.docx]

| **Adverse events** | **Transvenous pacemaker N = 267** | **Leadless  pacemaker N = 117** |
| --- | --- | --- |
| **Acute (< 90 days)** |  |  |
| Cardiac arrest n(%) | 13 (4.8) | 4 (3.4) |
| Hemopericardium n(%) | 0 (0) | 2 (1.7) |
| Pneumothorax /Hemothorax n(%) | 1 (0.1) | 0 (0) |
| Hemorrhage n(%) | 18 (6.7) | 7 (6.0) |
| Complication at the site of  access (pacemaker  implantation) n(%) | 11 (4.1) | 10 (8.5) |
| DVT/PE n(%) | 11 (4.1) | 5 (4.3) |
| Endocarditis/ Device related infection n(%) | 9 (3.4) | 2 (1.7) |
|  |  |  |
| **Long term** |  |  |
| Endocarditis/ Device related infection n(%) | 17 (6.3) | 3 (2.6) |
| DVT/PE n(%) | 37 (13.9) | 11 (9.4) |
|  |  |  |
| **Intervention on hemodialysis  vascular access** |  |  |
| At least one n(%) | 101 (37) | 26 (22) |
| Total number of interventions (n) | 221 | 48 |
| Time of exposure (py) | 296.32 | 85.82 |
| Annual rate (n/10 py, RR) | 7.46 | 5.59 |
| Type of intervention (n/10 py, RR) |  |  |
| First AVF creation | 0.47 | 0.35 |
| Ulterior AVF creation | 0.47 | 0.70 |
| Thrombectomy/angioplasty | 6.31 | 4.08 |
| AVF closure / Aneurysm resection | 0.20 | 0.47 |

**Table S3.** **Description of acute and long-term complications and interventions on vascular access in the whole wohort.** Statistical comparison was not performed as population were not comparable as baseline. py=person-years, DVT/PE= deep vein thrombosis/pulmonary embolism, AVF = arteriovenous fistula.
