# Supplementary material for: Improved outcomes with leadless vs. single-chamber transvenous pacemaker in haemodialysis patients
Source: Europace. 2024 Oct 1;26(11):euae257. doi: 10.1093/europace/euae257 (PMC11542626; doi:10.1093/europace/euae257)
Supplement: euae257_Supplementary_Data [file euae257_supplementary_data.zip › Table_S4_Description_infected_patients_2.docx]

| Patient | Sex | Age  (years) | BMI  (Kg/m2) | Diabetes | Vascular access  for hemodialysis | Time before diagnosis of DRI (in days) | Death | Time from infection to death (in days) |
| --- | --- | --- | --- | --- | --- | --- | --- | --- |
| 1 | M | 57 | 19.0 | YES | Arteriovenous fistula | 7 | NO | / |
| 2 | M | 85 | 21.1 | YES | Catheter | 835 | YES | 809 |
| 3 | M | 68 | 26.6 | YES | Arteriovenous fistula | 172 | YES | 621 |
| 4 | M | 63 | 34.6 | YES | Arteriovenous fistula | 45 | YES | 47 |
| 5 | M | 77 | 29.1 | YES | Arteriovenous fistula | 164 | YES | 20 |
| 6 | F | 74 | 16.5 | YES | Arteriovenous fistula | 108 | YES | 440 |
| 7 | F | 84 | 23.8 | NO | Arteriovenous fistula | 0 | YES | 0 |
| 8 | F | 86 | 40.6 | YES | Catheter | 0 | YES | 1004 |

**Table S4. Description of each patient with device related infection after single-chamber transvenous pacemaker implantation**. DVI= Device Related Infection.
