# Supplementary figures and images for: Improved outcomes with leadless vs. single-chamber transvenous pacemaker in haemodialysis patients
Source: Europace. 2024 Oct 1;26(11):euae257. doi: 10.1093/europace/euae257 (PMC11542626; doi:10.1093/europace/euae257)

# Covariate Balance

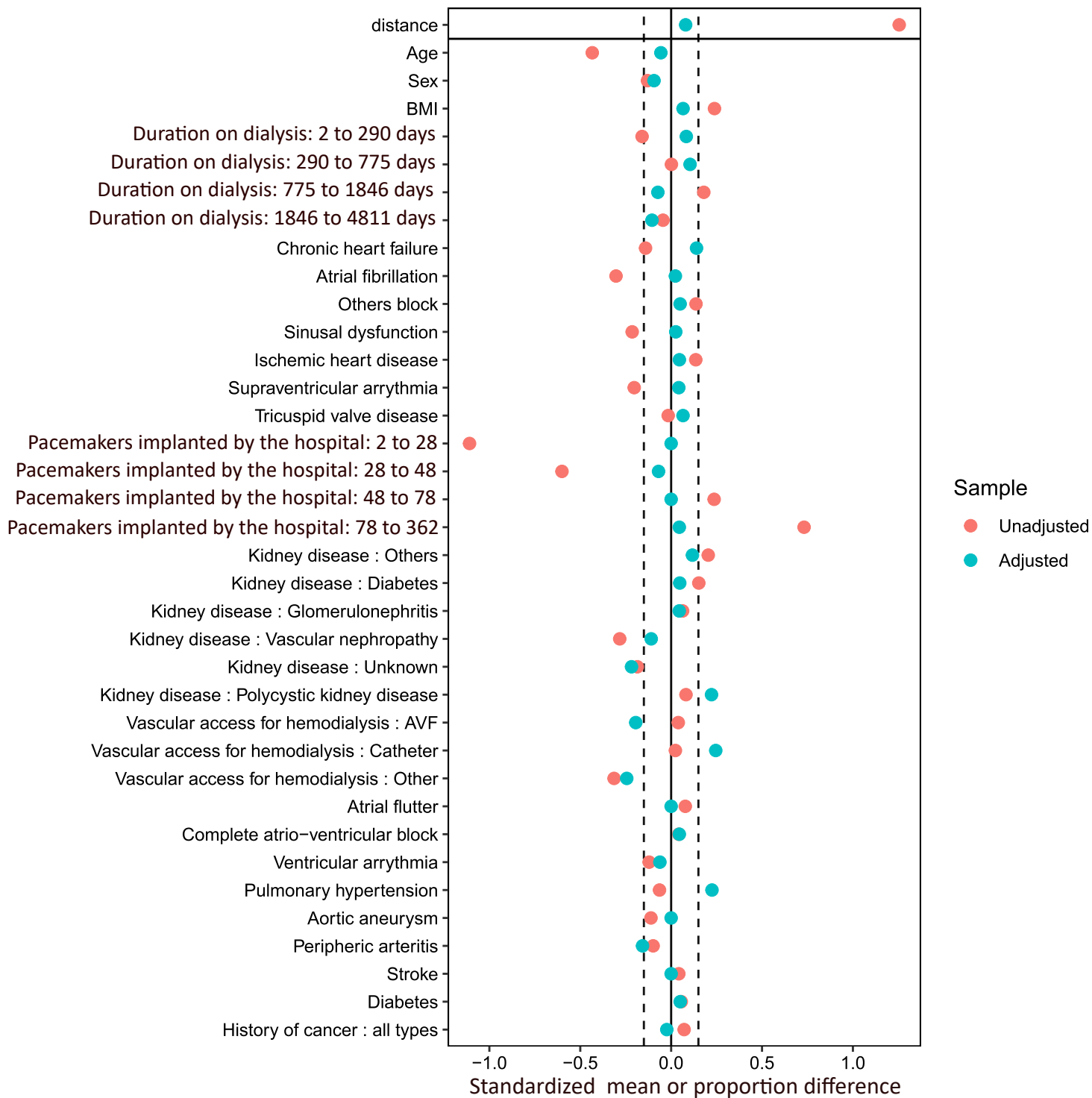

Supplement: euae257_Supplementary_Data [file euae257_supplementary_data.zip › Figure_S1_plot_matching_modified.pdf]

# Covariate Balance

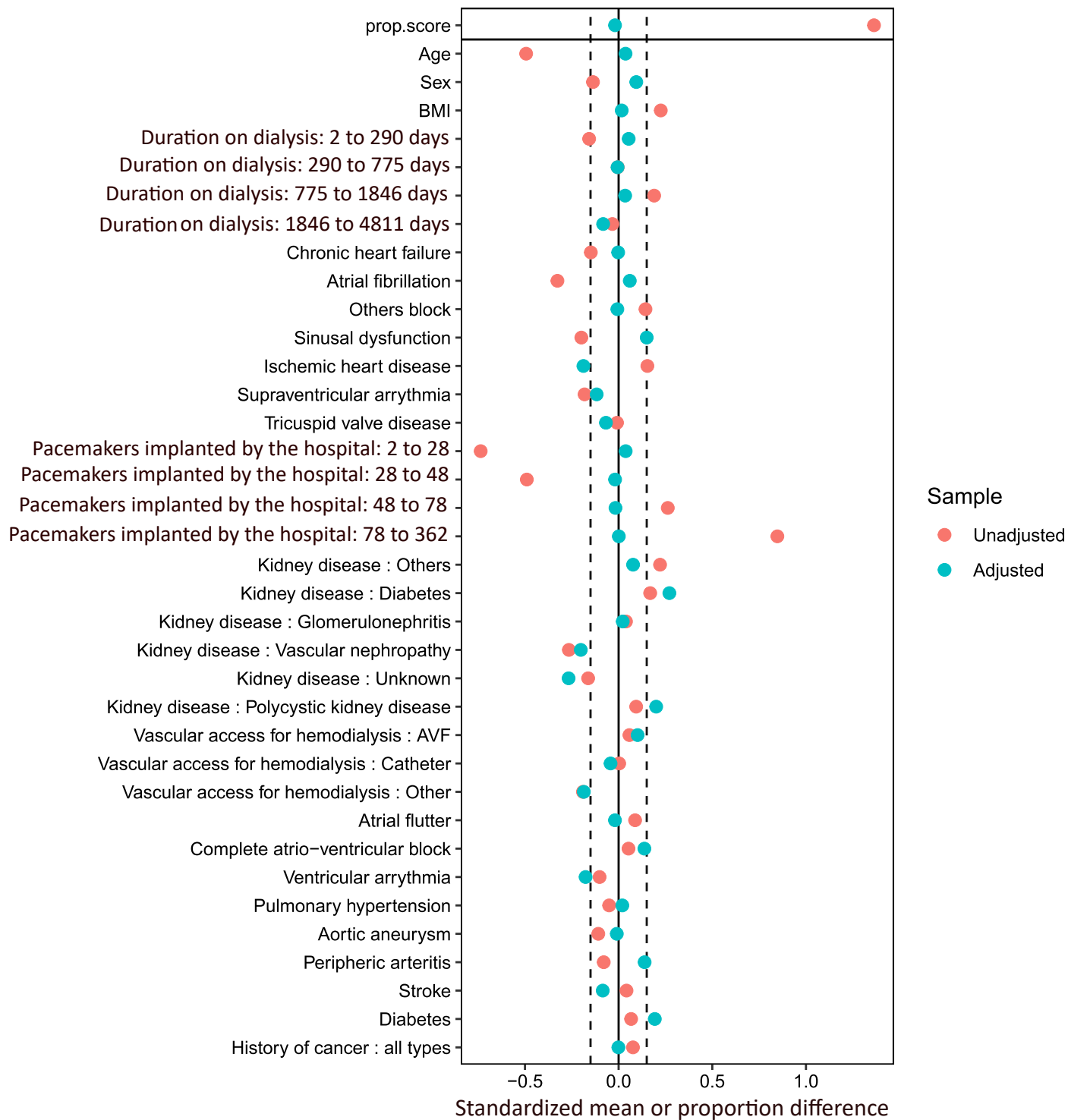

Supplement: euae257_Supplementary_Data [file euae257_supplementary_data.zip › Figure_S2_plot_weighting_modified.pdf]
